# *cis*-Dichlorido[2,3-dimethyl-3-(4,4,5,5-tetra­methyl-1,3,2λ^5^-dioxaphospho­lan-2-yl­oxy)butan-2-olato-κ^2^ *O*,*P*]oxido(triphenyl­phosphane-κ*P*)rhenium(V)

**DOI:** 10.1107/S1600536812015565

**Published:** 2012-04-18

**Authors:** Anna Skarżyńska, Miłosz Siczek, Andrzej Gniewek

**Affiliations:** a Faculty of Chemistry, University of Wrocław, 14 F. Joliot-Curie, 50-383 Wrocław, Poland

## Abstract

The title compound, *cis*-[Re(C_12_H_24_O_4_P)Cl_2_O(C_18_H_15_P)], was prepared from the analogous *trans* isomer [Głowiak *et al.* (2000 ▶). *Polyhedron*, **19**, 2667–2672] by a *trans*–*cis* isomerization reaction. The Re^V^ atom adopts a distorted octa­hedral coordination geometry. Besides being coordinated by the oxide and the butano­late O atoms, the Re^V^ atom is coordinated by a pair of chloride ligands and two P atoms in *cis* positions with respect to each other. In the crystal, adjacent mol­ecules are linked by weak C—H⋯Cl inter­actions, forming a three-dimensional network.

## Related literature

For related structures and further discussion, see: Głowiak *et al.* (1998 ▶, 2000 ▶); Rybak *et al.* (2005 ▶). For typical bond lengths in coordination complexes, see: Orpen *et al.* (1989 ▶). For hydrogen-bond inter­actions, see: Aullón *et al.* (1998 ▶); Desiraju & Steiner (1999 ▶); Fábry *et al.* (2004 ▶). For details of the temperature control unit used during the data collection, see: Cosier & Glazer (1986 ▶). For specifications of the analytical numeric absorption correction, see: Clark & Reid (1995 ▶).
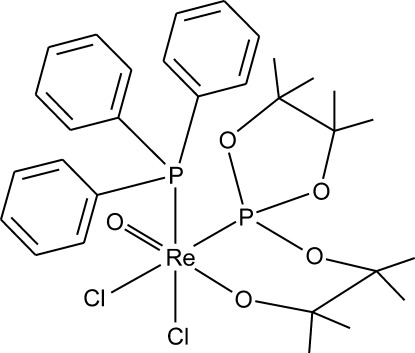


## Experimental

### 

#### Crystal data

[Re(C_12_H_24_O_4_P)Cl_2_O(C_18_H_15_P)]
*M* *_r_* = 798.65
Orthorhombic, 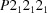

*a* = 10.963 (3) Å
*b* = 16.328 (4) Å
*c* = 17.797 (5) Å
*V* = 3185.7 (15) Å^3^
*Z* = 4
Mo *K*α radiation
μ = 4.12 mm^−1^
*T* = 100 K
0.16 × 0.12 × 0.05 mm

#### Data collection

Oxford Diffraction Xcalibur PX diffractometer with a CCD detector
Absorption correction: analytical (*CrysAlis RED*; Oxford Diffraction, 2010 ▶) *T*_min_ = 0.582, *T*_max_ = 0.808
15943 measured reflections
8614 independent reflections
6813 reflections with *I* > 2σ(*I*)
*R*_int_ = 0.036

#### Refinement

*R*[*F* ^2^ > 2σ(*F* ^2^)] = 0.026
*wR*(*F* ^2^) = 0.046
*S* = 0.87
8614 reflections
369 parameters
H-atom parameters constrained
Δρ_max_ = 1.21 e Å^−3^
Δρ_min_ = −1.09 e Å^−3^
Absolute structure: Flack (1983 ▶), 3463 Friedel pairs
Flack parameter: −0.015 (4)

### 

Data collection: *CrysAlis CCD* (Oxford Diffraction, 2010 ▶); cell refinement: *CrysAlis CCD*; data reduction: *CrysAlis RED* (Oxford Diffraction, 2010 ▶); program(s) used to solve structure: *SHELXS97* (Sheldrick, 2008 ▶); program(s) used to refine structure: *SHELXL97* (Sheldrick, 2008 ▶); molecular graphics: *ORTEP-3* (Farrugia, 1997 ▶); software used to prepare material for publication: *SHELXL97*.

## Supplementary Material

Crystal structure: contains datablock(s) global, I. DOI: 10.1107/S1600536812015565/su2403sup1.cif

Structure factors: contains datablock(s) I. DOI: 10.1107/S1600536812015565/su2403Isup2.hkl

Additional supplementary materials:  crystallographic information; 3D view; checkCIF report

## Figures and Tables

**Table 1:** Selected bond lengths (Å)

Re1—O1	1.698 (2)
Re1—O2	1.877 (3)
Re1—P1	2.3659 (12)
Re1—P2	2.4883 (12)
Re1—Cl1	2.4461 (10)
Re1—Cl2	2.4339 (10)

**Table 2:** Hydrogen-bond geometry (Å, °)

*D*—H⋯*A*	*D*—H	H⋯*A*	*D*⋯*A*	*D*—H⋯*A*
C31—H31*B*⋯Cl2^i^	0.98	2.86	3.796 (4)	161
C42—H42*B*⋯Cl1^ii^	0.98	2.87	3.830 (4)	167
C51—H51*B*⋯Cl1^ii^	0.98	2.85	3.809 (4)	166
C65—H65⋯Cl2^iii^	0.95	2.91	3.514 (4)	123

Symmetry codes: (i) 
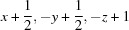
; (ii) 
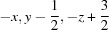
; (iii) 
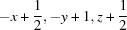
.

**Fig. 1.**
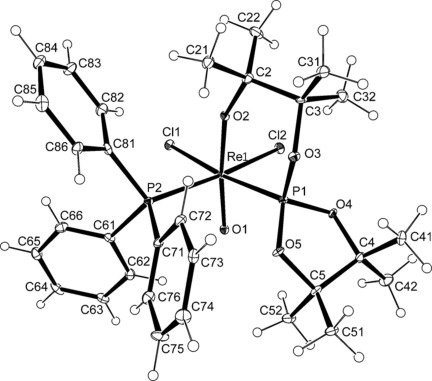
The molecular structure of the title compound, with atom numbering. Displacement ellipsoids are drawn at the 30% probability level.

## supplementary crystallographic information

### Comment

Most of the transition metal derivatives of spirophosphoranes are obtained in ligand substitution reactions, resulting in corresponding metal complexes with κ^2^-*O*,*P* ligand coordination mode (Rybak *et al.*, 2005). In this paper we report the synthesis and crystal structure of the title oxidorhenium(V) complex, obtained starting from an analogous *trans* complex (Głowiak *et al.* 2000). As a result of the *trans*-*cis* isomerization reaction single crystals of the *cis* isomer were obtained.

The coordination environment around the metal center, Re1, is a distorted octahedron with three sets of donor atoms: two O atoms in a *trans* arrangement and two chlorides and both phosphorus located in *cis* positions to each other (Fig. 1). The Re-ligand bond distances (Table 1) are generally similar to those reported for other rhenium complexes, nevertheless some disparities are observed. The distortions of the angles in the coordination sphere of the Re1 atom are significant, for example the O1—Re1—O2 angle of 168.36 (10) ° that differs from the expected value of 180°. The rhenium atom is located 0.06 Å out of the P1/P2/Cl1/Cl2 plane, towards the terminal oxo ligand. The Re1—P2 (phosphane) bond length of 2.4883 (12) Å is within the range 2.42–2.57 Å reported for analogous P*R*_3_ derivatives, however the Re1—P1 (phosphite) distance of 2.3659 (12) Å is quite short. This shortening may be explained by the strong π-acceptor character of the phosphite moiety and is consistent with the Re—P distances observed for other phosphite derivatives (Głowiak *et al.* 1998). The Re1—Cl bond lengths [2.4461 (10) and 2.4339 (10) Å] appear long compared with the expected values of 2.36–2.41 Å (Orpen *et al.*, 1989). This is a result of the high *trans* influence of the phosphorus ligands.

The crystal structure of the title compound is stabilized by a number of weak hydrogen bonds of the C—H···Cl type (Desiraju & Steiner, 1999). Consequently, a three-dimensional network is formed (Table 2). Even though the observed H···Cl distances may first appear to be fairly long compared with the expected values (Aullón *et al.*, 1998), the presence of C—H···Cl hydrogen bonds was confirmed spectroscopically for complexes with H···Cl spacings even above 3 Å (Fábry *et al.*, 2004).

### Experimental

The title compound, *cis*-[ReOCl_2_{P(OCMe_2_CMe_2_O)OCMe_2_CMe_2_O}PPh_3_], was prepared from an analogous *trans* isomer, which had been synthesized according to a previously reported procedure (Głowiak *et al.* 2000). The *trans* complex (0.1 g, 0.12 mmol) was dissolved in acetonitrile and refluxed for 6 h. Single crystals of the *cis* isomer suitable for the X-ray analysis were obtained after continuous, slow evaporation of the solvent at ambient temperature. Analysis for [C_30_H_39_Cl_2_O_5_P_2_Re]: calc. C 45.11, H 4.92; found: C 45.00, H 4.94%. Spectrosopic data for the title compound is given in the archived CIF.

### Refinement

The C-bonded H atoms were positioned geometrically and refined using a riding model: C—H = 0.95 and 0.98 Å for CH and CH_3_ H atoms, respectively, with *U*_iso_(H) = k × *U*_eq_(C), where k = 1.5 for CH_3_ H atoms, and = 1.2 for other H atoms. In the final difference electron density map the highest residual peak and the deepest hole are located 1.27 and 1.36 Å, respectively, from atom P2.

### Crystal data

**Table:**

[Re(C_12_H_24_O_4_P)Cl_2_O(C_18_H_15_P)]	*F*(000) = 1592
*M**_r_* = 798.65	*D*_x_ = 1.665 Mg m^−^^3^
Orthorhombic, *P*2_1_2_1_2_1_	Mo *K*α radiation, λ = 0.71073 Å
Hall symbol: P 2ac 2ab	Cell parameters from 8908 reflections
*a* = 10.963 (3) Å	θ = 4.8–30.0°
*b* = 16.328 (4) Å	µ = 4.12 mm^−^^1^
*c* = 17.797 (5) Å	*T* = 100 K
*V* = 3185.7 (15) Å^3^	Plate, orange
*Z* = 4	0.16 × 0.12 × 0.05 mm

### Data collection

**Table:**

Oxford Diffraction Xcalibur PX diffractometer with a CCD detector	8614 independent reflections
Radiation source: fine-focus sealed tube	6813 reflections with *I* > 2σ(*I*)
Graphite monochromator	*R*_int_ = 0.036
φ and ω scans	θ_max_ = 30.0°, θ_min_ = 4.8°
Absorption correction: analytical (*CrysAlis RED*; Oxford Diffraction, 2010)	*h* = −14→10
*T*_min_ = 0.582, *T*_max_ = 0.808	*k* = −22→15
15943 measured reflections	*l* = −24→23

### Refinement

**Table:**

Refinement on *F*^2^	Secondary atom site location: difference Fourier map
Least-squares matrix: full	Hydrogen site location: inferred from neighbouring sites
*R*[*F*^2^ > 2σ(*F*^2^)] = 0.026	H-atom parameters constrained
*wR*(*F*^2^) = 0.046	*w* = 1/[σ^2^(*F*_o_^2^) + (0.0111*P*)^2^] where *P* = (*F*_o_^2^ + 2*F*_c_^2^)/3
*S* = 0.87	(Δ/σ)_max_ = 0.002
8614 reflections	Δρ_max_ = 1.21 e Å^−^^3^
369 parameters	Δρ_min_ = −1.09 e Å^−^^3^
0 restraints	Absolute structure: Flack (1983), 3463 Friedel pairs
Primary atom site location: heavy-atom method	Flack parameter: −0.015 (4)

### Special details

**Table:**

Experimental. Spectrosopic data for the title compound: IR (KBr, cm^-1^): ν(C—O—P) 926 (*vs*), 941 (*vs*), 1019 (*s*), 1140 (*s*), ν(Re=O) 955 (*vs*). ^1^H NMR (CDCl_3_): δ 0.59, 0.82, 1.18, 1.28, 1.33, 1.43, 1.45, 1.54 (8*H*, s's, CH_3_), 7.46 (2*H*, m, CH), 7.52 (1*H*, m, CH), 7.76 (2*H*, m, CH). ^31^P NMR (CDCl_3_): δ -3.67, 87.4 p.p.m..The crystal was placed in the cold stream of an open-flow nitrogen cryostat (Cosier & Glazer, 1986) operating at 100 K. An analytical numeric absorption correction was carried out with *CrysAlis RED* (Oxford Diffraction, 2010) using a multifaceted crystal model (Clark & Reid, 1995).
Geometry. All e.s.d.'s (except the e.s.d. in the dihedral angle between two l.s. planes) are estimated using the full covariance matrix. The cell e.s.d.'s are taken into account individually in the estimation of e.s.d.'s in distances, angles and torsion angles; correlations between e.s.d.'s in cell parameters are only used when they are defined by crystal symmetry. An approximate (isotropic) treatment of cell e.s.d.'s is used for estimating e.s.d.'s involving l.s. planes.
Refinement. Refinement of *F*^2^ against ALL reflections. The weighted *R*-factor *wR* and goodness of fit *S* are based on *F*^2^, conventional *R*-factors *R* are based on *F*, with *F* set to zero for negative *F*^2^. The threshold expression of *F*^2^ > 2σ(*F*^2^) is used only for calculating *R*-factors(gt) *etc*. and is not relevant to the choice of reflections for refinement. *R*-factors based on *F*^2^ are statistically about twice as large as those based on *F* and *R*- factors based on ALL data will be even larger.

### Fractional atomic coordinates and isotropic or equivalent isotropic displacement parameters (Å^2^)

**Table:**

	*x*	*y*	*z*	*U*_iso_*/*U*_eq_	
Re1	0.24960 (2)	0.319412 (7)	0.721741 (7)	0.00859 (3)	
Cl1	0.30747 (8)	0.46317 (5)	0.73671 (5)	0.0139 (2)	
Cl2	0.15281 (9)	0.36291 (5)	0.60531 (6)	0.0153 (2)	
P1	0.20198 (9)	0.18153 (6)	0.69328 (6)	0.01015 (18)	
P2	0.35706 (9)	0.28754 (5)	0.84137 (6)	0.0099 (2)	
O1	0.1159 (2)	0.32297 (13)	0.76977 (15)	0.0121 (5)	
O2	0.3915 (2)	0.29251 (13)	0.66814 (15)	0.0108 (6)	
O3	0.3151 (2)	0.13616 (13)	0.65569 (16)	0.0127 (6)	
O4	0.0825 (2)	0.17154 (14)	0.64371 (15)	0.0120 (6)	
O5	0.1665 (2)	0.11895 (13)	0.75739 (15)	0.0120 (6)	
C2	0.4648 (4)	0.2422 (2)	0.6201 (2)	0.0138 (9)	
C21	0.5790 (3)	0.2159 (2)	0.6628 (3)	0.0178 (9)	
H21A	0.6164	0.2639	0.6865	0.027*	
H21B	0.6371	0.1911	0.6277	0.027*	
H21C	0.5568	0.1759	0.7015	0.027*	
C22	0.5024 (4)	0.2971 (2)	0.5541 (2)	0.0221 (10)	
H22A	0.4295	0.3216	0.5315	0.033*	
H22B	0.5453	0.2642	0.5164	0.033*	
H22C	0.5565	0.3406	0.5723	0.033*	
C3	0.3877 (3)	0.1669 (2)	0.5924 (2)	0.0119 (8)	
C31	0.4656 (4)	0.0946 (2)	0.5698 (2)	0.0189 (9)	
H31A	0.5057	0.0719	0.6144	0.028*	
H31B	0.5275	0.1124	0.5337	0.028*	
H31C	0.4140	0.0525	0.5468	0.028*	
C32	0.3019 (4)	0.1889 (2)	0.5283 (2)	0.0185 (9)	
H32A	0.2460	0.1432	0.5190	0.028*	
H32B	0.3496	0.1999	0.4828	0.028*	
H32C	0.2550	0.2378	0.5418	0.028*	
C4	0.0126 (4)	0.0977 (2)	0.6663 (2)	0.0137 (8)	
C41	0.0616 (4)	0.0267 (2)	0.6204 (3)	0.0211 (10)	
H41A	0.0612	0.0415	0.5670	0.032*	
H41B	0.0100	−0.0215	0.6283	0.032*	
H41C	0.1452	0.0144	0.6363	0.032*	
C42	−0.1203 (4)	0.1148 (2)	0.6464 (3)	0.0212 (10)	
H42A	−0.1444	0.1681	0.6670	0.032*	
H42B	−0.1722	0.0719	0.6678	0.032*	
H42C	−0.1297	0.1155	0.5916	0.032*	
C5	0.0380 (3)	0.0919 (2)	0.7508 (2)	0.0123 (8)	
C51	0.0327 (3)	0.00532 (19)	0.7833 (3)	0.0152 (8)	
H51A	0.0898	−0.0300	0.7561	0.023*	
H51B	−0.0503	−0.0163	0.7781	0.023*	
H51C	0.0550	0.0068	0.8366	0.023*	
C52	−0.0384 (4)	0.1492 (2)	0.7990 (2)	0.0182 (9)	
H52A	−0.0049	0.1509	0.8500	0.027*	
H52B	−0.1227	0.1293	0.8007	0.027*	
H52C	−0.0368	0.2043	0.7772	0.027*	
C61	0.3018 (5)	0.3519 (3)	0.9191 (3)	0.0120 (10)	
C62	0.1749 (4)	0.3585 (3)	0.9323 (3)	0.0151 (11)	
H62	0.1184	0.3309	0.9007	0.018*	
C63	0.1342 (4)	0.4053 (2)	0.9912 (3)	0.0158 (10)	
H63	0.0492	0.4087	1.0011	0.019*	
C64	0.2152 (4)	0.4477 (2)	1.0365 (3)	0.0204 (13)	
H64	0.1863	0.4802	1.0770	0.024*	
C65	0.3385 (4)	0.4421 (2)	1.0219 (3)	0.0188 (10)	
H65	0.3943	0.4717	1.0524	0.023*	
C66	0.3823 (4)	0.3943 (2)	0.9639 (3)	0.0172 (11)	
H66	0.4676	0.3907	0.9550	0.021*	
C71	0.3430 (3)	0.1828 (2)	0.8771 (2)	0.0112 (7)	
C72	0.4035 (3)	0.1196 (2)	0.8402 (2)	0.0146 (8)	
H72	0.4541	0.1316	0.7983	0.018*	
C73	0.3906 (4)	0.0390 (2)	0.8641 (2)	0.0140 (8)	
H73	0.4335	−0.0037	0.8394	0.017*	
C74	0.3157 (4)	0.0214 (2)	0.9236 (3)	0.0216 (10)	
H74	0.3058	−0.0338	0.9392	0.026*	
C75	0.2541 (6)	0.08333 (18)	0.9614 (2)	0.0217 (8)	
H75	0.2025	0.0707	1.0027	0.026*	
C76	0.2690 (4)	0.16424 (19)	0.9379 (2)	0.0168 (10)	
H76	0.2279	0.2070	0.9637	0.020*	
C81	0.5210 (3)	0.3057 (2)	0.8386 (2)	0.0130 (8)	
C82	0.5652 (4)	0.3742 (2)	0.8016 (2)	0.0176 (9)	
H82	0.5099	0.4106	0.7776	0.021*	
C83	0.6886 (4)	0.3902 (2)	0.7994 (2)	0.0198 (10)	
H83	0.7176	0.4376	0.7740	0.024*	
C84	0.7713 (4)	0.33728 (19)	0.8340 (2)	0.0187 (10)	
H84	0.8564	0.3475	0.8312	0.022*	
C85	0.7275 (4)	0.2695 (2)	0.8726 (2)	0.0186 (11)	
H85	0.7827	0.2335	0.8972	0.022*	
C86	0.6027 (4)	0.2543 (2)	0.8753 (2)	0.0147 (9)	
H86	0.5730	0.2084	0.9026	0.018*	

### Atomic displacement parameters (Å^2^)

**Table:**

	*U*^11^	*U*^22^	*U*^33^	*U*^12^	*U*^13^	*U*^23^
Re1	0.00898 (5)	0.00806 (5)	0.00874 (5)	0.00034 (11)	0.00038 (11)	0.00042 (6)
Cl1	0.0147 (4)	0.0095 (4)	0.0175 (6)	−0.0004 (4)	−0.0008 (4)	−0.0002 (4)
Cl2	0.0188 (5)	0.0149 (4)	0.0123 (5)	0.0020 (4)	−0.0035 (4)	0.0022 (4)
P1	0.0096 (4)	0.0098 (4)	0.0111 (5)	0.0000 (4)	0.0007 (4)	0.0001 (4)
P2	0.0103 (5)	0.0090 (4)	0.0103 (5)	−0.0007 (4)	0.0009 (4)	0.0005 (4)
O1	0.0119 (12)	0.0116 (11)	0.0128 (14)	0.0021 (11)	−0.0004 (11)	−0.0025 (12)
O2	0.0115 (13)	0.0099 (11)	0.0109 (15)	0.0009 (10)	0.0006 (12)	−0.0020 (11)
O3	0.0121 (14)	0.0088 (11)	0.0172 (16)	0.0009 (11)	0.0043 (12)	−0.0028 (11)
O4	0.0118 (13)	0.0113 (12)	0.0127 (15)	−0.0056 (11)	−0.0016 (11)	−0.0002 (11)
O5	0.0073 (13)	0.0110 (12)	0.0177 (16)	−0.0029 (10)	0.0002 (11)	0.0031 (10)
C2	0.0093 (19)	0.0145 (19)	0.018 (2)	0.0020 (16)	0.0008 (16)	−0.0021 (16)
C21	0.013 (2)	0.0197 (19)	0.021 (2)	0.0000 (17)	−0.0012 (18)	−0.0044 (18)
C22	0.032 (3)	0.019 (2)	0.015 (2)	−0.0042 (19)	0.008 (2)	−0.0013 (17)
C3	0.0088 (18)	0.012 (2)	0.014 (2)	−0.0009 (15)	0.0063 (15)	−0.0008 (16)
C31	0.017 (2)	0.019 (2)	0.021 (3)	−0.0040 (18)	0.0053 (19)	−0.0056 (18)
C32	0.0186 (19)	0.0193 (19)	0.018 (2)	0.0014 (19)	0.0002 (17)	−0.0032 (18)
C4	0.015 (2)	0.0105 (17)	0.015 (2)	−0.0044 (16)	−0.0013 (18)	0.0012 (17)
C41	0.025 (2)	0.017 (2)	0.021 (3)	−0.0075 (19)	0.000 (2)	−0.0085 (18)
C42	0.011 (2)	0.027 (2)	0.025 (3)	−0.0013 (18)	−0.0033 (19)	0.0050 (19)
C5	0.0083 (18)	0.0106 (17)	0.018 (2)	−0.0027 (15)	−0.0002 (16)	0.0013 (16)
C51	0.0129 (18)	0.0144 (17)	0.018 (2)	−0.0037 (15)	0.0045 (19)	0.0018 (18)
C52	0.020 (2)	0.0138 (18)	0.020 (3)	−0.0009 (17)	0.0081 (18)	0.0010 (16)
C61	0.017 (2)	0.0074 (19)	0.011 (2)	0.0015 (17)	0.0019 (19)	0.0020 (16)
C62	0.016 (2)	0.014 (2)	0.015 (3)	0.001 (2)	−0.003 (2)	0.0004 (19)
C63	0.019 (2)	0.015 (2)	0.014 (3)	0.0072 (19)	0.003 (2)	0.0048 (19)
C64	0.042 (4)	0.0092 (17)	0.010 (2)	0.0057 (18)	0.0033 (19)	−0.0015 (15)
C65	0.028 (3)	0.013 (2)	0.016 (3)	−0.003 (2)	−0.003 (2)	−0.0015 (18)
C66	0.017 (2)	0.013 (2)	0.022 (3)	−0.0013 (18)	0.004 (2)	−0.0001 (19)
C71	0.0128 (18)	0.0075 (15)	0.013 (2)	−0.0026 (17)	−0.0013 (15)	0.0009 (17)
C72	0.0113 (19)	0.0163 (19)	0.016 (2)	−0.0032 (16)	−0.0008 (17)	0.0007 (17)
C73	0.016 (2)	0.0116 (17)	0.015 (2)	−0.0005 (17)	−0.0046 (17)	−0.0018 (16)
C74	0.030 (3)	0.013 (2)	0.021 (3)	−0.0036 (18)	−0.004 (2)	0.0073 (17)
C75	0.030 (2)	0.0160 (15)	0.019 (2)	−0.002 (3)	0.008 (3)	0.0074 (14)
C76	0.017 (3)	0.0129 (17)	0.021 (2)	0.0001 (16)	−0.0001 (17)	−0.0020 (13)
C81	0.0172 (19)	0.0130 (19)	0.009 (2)	0.0002 (16)	−0.0014 (16)	−0.0031 (16)
C82	0.016 (2)	0.0163 (19)	0.021 (2)	0.0007 (17)	−0.0018 (17)	−0.0005 (17)
C83	0.014 (2)	0.0195 (19)	0.026 (3)	−0.0048 (17)	−0.0013 (18)	0.0036 (17)
C84	0.012 (3)	0.0197 (18)	0.025 (2)	−0.0008 (15)	−0.0019 (17)	−0.0050 (15)
C85	0.019 (3)	0.0146 (16)	0.022 (2)	0.0078 (17)	−0.0052 (17)	−0.0044 (15)
C86	0.017 (2)	0.0096 (17)	0.018 (2)	−0.0048 (16)	−0.0029 (17)	0.0017 (16)

### Geometric parameters (Å, º)

**Table:**

Re1—O1	1.698 (2)	C5—C52	1.520 (5)
Re1—O2	1.877 (3)	C5—C51	1.529 (5)
Re1—P1	2.3659 (12)	C51—H51A	0.9800
Re1—P2	2.4883 (12)	C51—H51B	0.9800
Re1—Cl1	2.4461 (10)	C51—H51C	0.9800
Re1—Cl2	2.4339 (10)	C52—H52A	0.9800
P1—O3	1.592 (3)	C52—H52B	0.9800
P1—O4	1.588 (3)	C52—H52C	0.9800
P1—O5	1.580 (3)	C61—C66	1.377 (6)
P2—C61	1.840 (4)	C61—C62	1.415 (5)
P2—C71	1.831 (4)	C62—C63	1.372 (6)
P2—C81	1.822 (4)	C62—H62	0.9500
O2—C2	1.432 (4)	C63—C64	1.384 (6)
O3—C3	1.468 (4)	C63—H63	0.9500
O4—C4	1.484 (4)	C64—C65	1.380 (6)
O5—C5	1.481 (4)	C64—H64	0.9500
C2—C21	1.526 (5)	C65—C66	1.380 (6)
C2—C22	1.534 (5)	C65—H65	0.9500
C2—C3	1.571 (5)	C66—H66	0.9500
C21—H21A	0.9800	C71—C76	1.386 (5)
C21—H21B	0.9800	C71—C72	1.391 (5)
C21—H21C	0.9800	C72—C73	1.390 (5)
C22—H22A	0.9800	C72—H72	0.9500
C22—H22B	0.9800	C73—C74	1.369 (6)
C22—H22C	0.9800	C73—H73	0.9500
C3—C31	1.511 (5)	C74—C75	1.390 (6)
C3—C32	1.520 (5)	C74—H74	0.9500
C31—H31A	0.9800	C75—C76	1.395 (4)
C31—H31B	0.9800	C75—H75	0.9500
C31—H31C	0.9800	C76—H76	0.9500
C32—H32A	0.9800	C81—C82	1.386 (5)
C32—H32B	0.9800	C81—C86	1.390 (5)
C32—H32C	0.9800	C82—C83	1.378 (5)
C4—C41	1.515 (5)	C82—H82	0.9500
C4—C42	1.525 (5)	C83—C84	1.396 (5)
C4—C5	1.534 (6)	C83—H83	0.9500
C41—H41A	0.9800	C84—C85	1.389 (5)
C41—H41B	0.9800	C84—H84	0.9500
C41—H41C	0.9800	C85—C86	1.391 (6)
C42—H42A	0.9800	C85—H85	0.9500
C42—H42B	0.9800	C86—H86	0.9500
C42—H42C	0.9800		
			
O1—Re1—O2	168.36 (10)	C4—C42—H42A	109.5
O1—Re1—P1	87.13 (8)	C4—C42—H42B	109.5
O2—Re1—P1	81.45 (8)	H42A—C42—H42B	109.5
O1—Re1—P2	89.15 (9)	C4—C42—H42C	109.5
O2—Re1—P2	89.63 (9)	H42A—C42—H42C	109.5
O1—Re1—Cl2	92.42 (9)	H42B—C42—H42C	109.5
O2—Re1—Cl2	89.82 (9)	O5—C5—C52	107.3 (3)
P1—Re1—Cl2	89.96 (3)	O5—C5—C51	106.4 (3)
O1—Re1—Cl1	97.83 (8)	C52—C5—C51	109.6 (3)
O2—Re1—Cl1	93.73 (8)	O5—C5—C4	103.4 (3)
P1—Re1—Cl1	173.56 (3)	C52—C5—C4	114.5 (3)
P2—Re1—Cl2	174.79 (3)	C51—C5—C4	114.9 (3)
Cl1—Re1—Cl2	85.74 (3)	C5—C51—H51A	109.5
P1—Re1—P2	95.09 (3)	C5—C51—H51B	109.5
Cl1—Re1—P2	89.12 (3)	H51A—C51—H51B	109.5
O5—P1—O4	97.57 (14)	C5—C51—H51C	109.5
O5—P1—O3	101.19 (13)	H51A—C51—H51C	109.5
O4—P1—O3	111.19 (15)	H51B—C51—H51C	109.5
O5—P1—Re1	121.00 (10)	C5—C52—H52A	109.5
O4—P1—Re1	113.50 (10)	C5—C52—H52B	109.5
O3—P1—Re1	111.16 (9)	H52A—C52—H52B	109.5
C81—P2—C71	104.11 (17)	C5—C52—H52C	109.5
C81—P2—C61	104.6 (2)	H52A—C52—H52C	109.5
C71—P2—C61	104.15 (19)	H52B—C52—H52C	109.5
C81—P2—Re1	114.21 (13)	C66—C61—C62	119.7 (5)
C71—P2—Re1	116.91 (12)	C66—C61—P2	120.8 (4)
C61—P2—Re1	111.59 (15)	C62—C61—P2	119.5 (4)
C2—O2—Re1	154.5 (2)	C63—C62—C61	119.3 (5)
C3—O3—P1	125.9 (2)	C63—C62—H62	120.4
C4—O4—P1	111.0 (2)	C61—C62—H62	120.4
C5—O5—P1	111.7 (2)	C62—C63—C64	121.0 (4)
O2—C2—C21	109.0 (3)	C62—C63—H63	119.5
O2—C2—C22	105.8 (3)	C64—C63—H63	119.5
C21—C2—C22	108.9 (3)	C65—C64—C63	119.0 (5)
O2—C2—C3	109.6 (3)	C65—C64—H64	120.5
C21—C2—C3	112.2 (3)	C63—C64—H64	120.5
C22—C2—C3	111.1 (3)	C64—C65—C66	121.3 (5)
C2—C21—H21A	109.5	C64—C65—H65	119.4
C2—C21—H21B	109.5	C66—C65—H65	119.4
H21A—C21—H21B	109.5	C61—C66—C65	119.7 (5)
C2—C21—H21C	109.5	C61—C66—H66	120.2
H21A—C21—H21C	109.5	C65—C66—H66	120.2
H21B—C21—H21C	109.5	C76—C71—C72	119.0 (3)
C2—C22—H22A	109.5	C76—C71—P2	121.6 (3)
C2—C22—H22B	109.5	C72—C71—P2	119.3 (3)
H22A—C22—H22B	109.5	C73—C72—C71	120.6 (4)
C2—C22—H22C	109.5	C73—C72—H72	119.7
H22A—C22—H22C	109.5	C71—C72—H72	119.7
H22B—C22—H22C	109.5	C74—C73—C72	119.8 (4)
O3—C3—C31	104.1 (3)	C74—C73—H73	120.1
O3—C3—C32	108.7 (3)	C72—C73—H73	120.1
C31—C3—C32	109.6 (3)	C73—C74—C75	120.8 (4)
O3—C3—C2	108.5 (3)	C73—C74—H74	119.6
C31—C3—C2	113.0 (3)	C75—C74—H74	119.6
C32—C3—C2	112.6 (3)	C74—C75—C76	119.1 (4)
C3—C31—H31A	109.5	C74—C75—H75	120.4
C3—C31—H31B	109.5	C76—C75—H75	120.4
H31A—C31—H31B	109.5	C71—C76—C75	120.7 (4)
C3—C31—H31C	109.5	C71—C76—H76	119.7
H31A—C31—H31C	109.5	C75—C76—H76	119.7
H31B—C31—H31C	109.5	C82—C81—C86	119.1 (3)
C3—C32—H32A	109.5	C82—C81—P2	119.2 (3)
C3—C32—H32B	109.5	C86—C81—P2	121.6 (3)
H32A—C32—H32B	109.5	C83—C82—C81	120.7 (4)
C3—C32—H32C	109.5	C83—C82—H82	119.7
H32A—C32—H32C	109.5	C81—C82—H82	119.7
H32B—C32—H32C	109.5	C82—C83—C84	120.5 (4)
O4—C4—C41	107.0 (3)	C82—C83—H83	119.8
O4—C4—C42	106.3 (3)	C84—C83—H83	119.8
C41—C4—C42	110.7 (3)	C85—C84—C83	119.2 (4)
O4—C4—C5	102.8 (3)	C85—C84—H84	120.4
C41—C4—C5	114.6 (3)	C83—C84—H84	120.4
C42—C4—C5	114.4 (4)	C84—C85—C86	119.9 (4)
C4—C41—H41A	109.5	C84—C85—H85	120.0
C4—C41—H41B	109.5	C86—C85—H85	120.0
H41A—C41—H41B	109.5	C81—C86—C85	120.6 (4)
C4—C41—H41C	109.5	C81—C86—H86	119.7
H41A—C41—H41C	109.5	C85—C86—H86	119.7
H41B—C41—H41C	109.5		
			
O1—RE1—P1—O5	−51.92 (14)	P1—O4—C4—C5	32.4 (3)
O2—RE1—P1—O5	125.82 (14)	P1—O5—C5—C52	−92.2 (3)
Cl2—RE1—P1—O5	−144.35 (12)	P1—O5—C5—C51	150.6 (2)
P2—RE1—P1—O5	36.96 (12)	P1—O5—C5—C4	29.1 (3)
O1—RE1—P1—O4	63.47 (14)	O4—C4—C5—O5	−36.4 (3)
O2—RE1—P1—O4	−118.78 (14)	C41—C4—C5—O5	79.3 (4)
Cl2—RE1—P1—O4	−28.96 (12)	C42—C4—C5—O5	−151.2 (3)
P2—RE1—P1—O4	152.35 (12)	O4—C4—C5—C52	79.9 (4)
O1—RE1—P1—O3	−170.31 (15)	C41—C4—C5—C52	−164.3 (3)
O2—RE1—P1—O3	7.44 (14)	C42—C4—C5—C52	−34.9 (4)
Cl2—RE1—P1—O3	97.26 (12)	O4—C4—C5—C51	−151.9 (3)
P2—RE1—P1—O3	−81.42 (12)	C41—C4—C5—C51	−36.2 (5)
O1—RE1—P2—C81	−161.66 (14)	C42—C4—C5—C51	93.2 (4)
O2—RE1—P2—C81	29.91 (14)	C81—P2—C61—C66	−5.3 (4)
P1—RE1—P2—C81	111.30 (12)	C71—P2—C61—C66	103.7 (4)
Cl1—RE1—P2—C81	−63.82 (12)	RE1—P2—C61—C66	−129.3 (3)
O1—RE1—P2—C71	76.47 (16)	C81—P2—C61—C62	173.9 (4)
O2—RE1—P2—C71	−91.95 (15)	C71—P2—C61—C62	−77.1 (5)
P1—RE1—P2—C71	−10.57 (14)	RE1—P2—C61—C62	49.9 (5)
Cl1—RE1—P2—C71	174.32 (14)	C66—C61—C62—C63	−2.0 (8)
O1—RE1—P2—C61	−43.24 (18)	P2—C61—C62—C63	178.8 (3)
O2—RE1—P2—C61	148.34 (17)	C61—C62—C63—C64	1.8 (7)
P1—RE1—P2—C61	−130.28 (16)	C62—C63—C64—C65	−0.3 (7)
Cl1—RE1—P2—C61	54.60 (16)	C63—C64—C65—C66	−0.9 (7)
O1—RE1—O2—C2	23.7 (10)	C62—C61—C66—C65	0.8 (7)
P1—RE1—O2—C2	12.4 (6)	P2—C61—C66—C65	−180.0 (3)
Cl2—RE1—O2—C2	−77.5 (6)	C64—C65—C66—C61	0.6 (7)
Cl1—RE1—O2—C2	−163.3 (6)	C81—P2—C71—C76	127.8 (3)
P2—RE1—O2—C2	107.6 (6)	C61—P2—C71—C76	18.4 (4)
O5—P1—O3—C3	−179.1 (3)	RE1—P2—C71—C76	−105.2 (3)
O4—P1—O3—C3	78.1 (3)	C81—P2—C71—C72	−55.3 (3)
RE1—P1—O3—C3	−49.4 (3)	C61—P2—C71—C72	−164.7 (3)
O5—P1—O4—C4	−14.6 (3)	RE1—P2—C71—C72	71.7 (3)
O3—P1—O4—C4	90.5 (2)	C76—C71—C72—C73	−0.4 (6)
RE1—P1—O4—C4	−143.3 (2)	P2—C71—C72—C73	−177.4 (3)
O4—P1—O5—C5	−9.6 (2)	C71—C72—C73—C74	1.3 (6)
O3—P1—O5—C5	−123.1 (2)	C72—C73—C74—C75	−1.2 (7)
RE1—P1—O5—C5	113.7 (2)	C73—C74—C75—C76	0.2 (8)
RE1—O2—C2—C21	−119.4 (5)	C72—C71—C76—C75	−0.6 (7)
RE1—O2—C2—C22	123.7 (5)	P2—C71—C76—C75	176.2 (4)
RE1—O2—C2—C3	3.8 (7)	C74—C75—C76—C71	0.7 (8)
P1—O3—C3—C31	−169.5 (2)	C71—P2—C81—C82	168.6 (3)
P1—O3—C3—C32	−52.8 (4)	C61—P2—C81—C82	−82.3 (4)
P1—O3—C3—C2	70.0 (4)	RE1—P2—C81—C82	40.0 (3)
O2—C2—C3—O3	−41.2 (4)	C71—P2—C81—C86	−14.1 (4)
C21—C2—C3—O3	80.0 (4)	C61—P2—C81—C86	94.9 (3)
C22—C2—C3—O3	−157.8 (3)	RE1—P2—C81—C86	−142.8 (3)
O2—C2—C3—C31	−156.1 (3)	C86—C81—C82—C83	1.8 (6)
C21—C2—C3—C31	−34.9 (5)	P2—C81—C82—C83	179.2 (3)
C22—C2—C3—C31	87.4 (4)	C81—C82—C83—C84	0.3 (7)
O2—C2—C3—C32	79.2 (4)	C82—C83—C84—C85	−1.8 (6)
C21—C2—C3—C32	−159.6 (3)	C83—C84—C85—C86	1.1 (6)
C22—C2—C3—C32	−37.4 (4)	C82—C81—C86—C85	−2.5 (6)
P1—O4—C4—C41	−88.7 (3)	P2—C81—C86—C85	−179.8 (3)
P1—O4—C4—C42	152.9 (3)	C84—C85—C86—C81	1.1 (6)

### Hydrogen-bond geometry (Å, º)

**Table:**

*D*—H···*A*	*D*—H	H···*A*	*D*···*A*	*D*—H···*A*
C31—H31*B*···Cl2^i^	0.98	2.86	3.796 (4)	161
C42—H42*B*···Cl1^ii^	0.98	2.87	3.830 (4)	167
C51—H51*B*···Cl1^ii^	0.98	2.85	3.809 (4)	166
C65—H65···Cl2^iii^	0.95	2.91	3.514 (4)	123

Symmetry codes: (i) *x*+1/2, −*y*+1/2, −*z*+1; (ii) −*x*, *y*−1/2, −*z*+3/2; (iii) −*x*+1/2, −*y*+1, *z*+1/2.
